# The importance of a large sample cohort for studies on modifier genes influencing disease severity in FAP patients

**DOI:** 10.1186/1897-4287-11-20

**Published:** 2013-12-29

**Authors:** Bente A Talseth-Palmer, Juul T Wijnen, Eva K Andreassen, Daniel Barker, Shantie Jagmohan-Changur, Carli M Tops, Cliff Meldrum, Allan Spigelman, Frederik J Hes, Tom Van Wezel, Hans FA Vasen, Rodney J Scott

**Affiliations:** 1 School of Biomedical Sciences and Pharmacy, University of Newcastle, Newcastle, Australia; 2 Hunter Medical Research Institute, John Hunter Hospital, Newcastle, Australia; 3 Department of Human and Department of Clinical Genetics, Leiden University Medical Centre, Leiden, the Netherlands; 4 Department of Pharmacology, The Institute of Pharmacy, Faculty of Medicine, University of Tromsø, Tromsø, Norway; 5 School of Medicine and Public Health, University of Newcastle, Newcastle, Australia; 6 Hunter Area Pathology Service, Hunter New England Area Health, Newcastle, Australia; 7 The Dutch Cancer Genetics Group, Leiden, the Netherlands; 8 Hunter Family Cancer Service, Hunter New England Area Health, Newcastle, Australia; 9 University of NSW, St Vincent’s Hospital Clinical School, Sydney, Australia; 10 Hereditary Cancer Clinic, St Vincent’s Hospital, The Kinghorn Cancer Centre, Sydney, Australia; 11 Department of Pathology, Leiden University Medical Centre, Leiden, the Netherlands; 12 Dutch Foundation for the Detection of Hereditary Tumours, Leiden, the Netherlands

**Keywords:** Modifier genes, Disease severity, FAP

## Abstract

**Background:**

Familial adenomatous polyposis (FAP) is usually characterised by the appearance of hundreds-to-thousands of adenomas throughout the colon and rectum and if left untreated the condition will develop into CRC with close to 100% penetrance. Germline mutations in the *APC* gene, which plays an integral role in the *Wnt*-signalling pathway, have been found to be responsible for 70-90% of FAP cases. Several studies suggest that modifier genes may play an important role in the development of CRC and possible modifiers for FAP have been suggested. Interestingly, a study has found that SNPs within *ATP5A1* is associated with raised levels of *ATP5A1* expression and high expression levels may facilitate CRC development. We aimed to determine if SNPs in *ATP5A1* modify the risk of developing CRC/adenomas in FAP patients.

**Methods:**

Genomic DNA from 139 Australian FAP patients with a germline *APC* mutation underwent genotyping at the Australian Genome Research Facility (AGRF) utilising iPLEX GOLD chemistry with Sequenom MassArray on an Autoflex Spectrometer for 16 SNPs in the *ATP5A1* gene. Association between ages of diagnosis/risk of CRC/adenomas was tested with Kaplan-Meier estimator analysis, logistic regression and cox proportional hazard regression.

**Results:**

An association between age of diagnosis of CRC and genotypes was observed for SNP rs2578189 (*p* = 0.0014), with individuals harbouring the variant genotype developing CRC 29 years earlier than individuals harbouring the wildtype genotype. Individuals harbouring the variant genotype of SNP rs2578189 were also at increased risk of CRC (HR = 13.79, 95% CI = 2.36-80.64, *p* = 0.004). We used an independent Dutch FAP cohort (n = 427) to validate our results; no association between SNP rs2578189 and CRC was observed.

**Conclusion:**

These results highlight the difficulties in studying a disease that has a high degree of intervention and also emphasize the importance of large sample sizes when searching for modifier genes in patients with an inherited predisposition to disease. To fully determine if there are genetic modifiers of disease in FAP we would encourage people that are interested in collaborating in future studies into the role of modifier genes in disease expression in FAP to join forces.

## Background

Colorectal cancer (CRC) is one of the most common and preventable forms of cancers worldwide, with an annual incidence of ~16,000 and accounting for ~4,000 deaths in Australia alone [1]. Several genetic and environmental factors contribute to the development of cancer and it is estimated that up to 35% of all CRCs are caused by a genetic predisposition [2]. Familial adenomatous polyposis (FAP) accounts for approximately 1% of all CRCs [3] and is an inherited autosomal-dominant condition characterised by the appearance of hundreds to thousands of adenomas throughout the colon and rectum [3]. The disease has almost 100% penetrance and if left untreated will ultimately develop into CRC by the third or fourth decade of life. Extra-colonic manifestation occur in approximately 70% of FAP patients [4] and include desmoid tumours, congenital hypertrophy of the retinal pigment epithelium (CHRPE), hepatoblastoma, fundic gland polyps, pancreatic and thyroid cancers, dental abnormalities and malignant tumours of the central nervous system [3,5,6].

Truncating germline mutations in the *adenomatous polyposis coli (APC)* gene are responsible for 70-90% of FAP cases [7]. *APC* plays an integral role in the Wnt-signalling pathway, especially in regards to the degradation of β-catenin within the cell cytoplasm. Considerable variability in disease expression is observed within families and among individuals with identical mutations exist [8] and it has been shown that the greater the number of colorectal adenomas, the greater the CRC risk is [9]. Even though haplotype reconstructions from pedigrees found no evidence for a specific *APC* haplotype associated with disease severity [10], genotype-phenotype correlations have been associated with the location of germline mutations within *APC* that are related to the severity of polyposis and expression of extra-colonic features [7,11]. Patients with mutations in the mutation cluster region (MCR), located between codons 1286 to 1513 [12], have generally a worse prognosis with earlier onset of disease [13]. Most severe disease is associated with germline mutations at codon 1309 [14], while milder forms of disease with less than 100 adenomas and later ages of onset (attenuated FAP (AFAP)) is associated with codons <157, 312–412 and >1595 [11,15].

Several studies suggest that low-penetrant susceptibility genes may play an important role in the development of sporadic CRC [16-21]. There is evidence to show that variation in FAP severity, which has been shown to be independent of *APC* mutation and most likely the action of modifier genes, is expected to result in different rates of tumour initiation (adenoma number) rather than differences in tumour progression i.e. adenomas to carcinoma [22]. Modifier genes can influence individual susceptibility to cancer by enhancing or supressing the initiation, growth and/or progression of tumour cells. The pattern of intrafamilial variation in colonic FAP severity is consistent with the action of modifier genes [10,22-24].

Interestingly, a study has found that the *ATP5A1* gene (chromosome 18q21) may act as a modifier gene in the development of CRC in the murine version of human FAP (*APC*^*Min*^ mice) [25]. The gene has previously been shown to suppress polyp formation in mice when it is mutated, with mutant Atp5a1 mice showing a reduction in small intestinal and colonic polyps by approximately 90% [26]. Unexpectedly, this mutation has also shown that more adenomas progress to carcinomas in *Min* mice that carry the Atp5a1 mutation [25]. The *ATP5A1* gene encodes the alpha-subunit of ATP synthase, a multi-subunit enzyme which resided on the mitochondrial membrane. Absence of *ATP5A1* leads to a non-functional α-synthesis subunit and subsequent apoptosis of the cell [27]. Raised levels of *ATP5A1* expression have been associated with certain single nucleotide polymorphisms (SNPs) and high expression levels together with chromosome instability (CIN) may facilitate CRC development [28]. This study aims to investigate if SNPs in the *ATP5A1* gene can act as modifiers for CRC development in FAP patients.

## Methods

### Sample cohort

Genomic DNA from 139 FAP patients from 86 families with a molecular diagnosis of FAP (carriers of germline *APC* mutations) was obtained from the Hunter Area Pathology Service (HAPS), John Hunter Hospital, Newcastle, New South Wales, Australia. All samples were collected between the years 1997 and 2008. Each participant (n = 139) had previously contributed blood from which DNA was extracted using the salt precipitation method [29]. All the participants in the study have given informed consent for their de-identified DNA to be used for future research into the cause of their condition. Ethics approval was obtained from Hunter Area Research Ethics Committee and the University of Newcastle Human Research Ethics Committee.

### Genotyping

The genotyping was outsourced to the Australian Genome Research Facility (AGRF) who utilised iPLEX GOLD chemistry with Sequenom MassArray on an Autoflex spectrometer to genotype 16 SNPs in *ATP5A1* (18q21); rs13381709, rs1800636, rs1800637, rs1800639, rs1800640, rs2298787, rs2578187, rs2578189, rs7244921, rs8088881, rs8089150, rs8092674, rs8093880, rs8094902, rs8095031 and rs8095608.

### Statistical analysis

A Pearson’s Chi-square test was used to evaluate deviation from the expected Hardy-Weinberg equilibrium (HWE). Fourteen of the sixteen SNPs seemed to be in linkage disequilibrium as 137/139 samples have the exact same genotypes; we are therefore only testing three SNPs and applied Bonferoni correction for multiple testing resulting in a corrected significance threshold of: *p* = 0.05/3 = 0.0167. Risk of CRC association with each SNP was estimated by heterozygous and homozygous odds ratio (OR) using simple logistic regression and multiple logistic regressions to adjust for gender (male; female) and mutation group according to severe (APC MCR = codons 1250–1513), attenuated (APC AFAP = codons <157, 312–412 and >1595) and intermediate (APC = the rest of the gene) polyposis phenotypes. Kaplan-Meier estimator analysis was used to test association between age of diagnosis of CRC and genotypes. Age of diagnoses of CRC were used as estimate functions, while age at last follow up was used for cancer free individuals. Wilcoxon’s (Breslow), Log-rank and Tarone-Ware tests were used to examine homogeneity of the Kaplan-Meier plots. All three tests were required to be significant for results to be considered reliable but only log-rank test is reported in Table 1. If a SNP was found to be associated with risk of CRC/adenomas through logistic regression/Kaplan-Meier, association between genotype and risk of CRC/adenomas was further evaluated using Cox proportional hazard model. Clustering of samples within families were adjusted for by including family as a frailty term in the model, if there was more than one member of a family, patients were grouped together as one ‘cluster’.

**Table 1:** Logistic regression and Kaplan-Meier analysis

**Logistic regression model (n = 139)**						**Analysis endpoint - CRC**			**Analysis endpoint - Adenomas**		
***APT5A1 *** **SNP: Genotype**		**Total n (%)**	**Cancer free (n)**	**CRC (n)**	**Adenomas (n)**	**OR (95% CI)**	**p-value**	Kaplan-Meier analysis p-value*	**OR (95% CI)**	**p-value**	Kaplan-Meier analysis p-value*
rs13381709:	CC	59 (42)	51	8	44	1.0		0.3183	1.0		0.2561
	CT	57 (41)	46	11	42	1.52 (0.56-4.12)	0.406		0.95 (0.42-2.19)	0.913	
	TT	23 (17)	22	1	13	0.29 (0.03-2.46)	0.256		0.44 (0.16-1.22)	0.115	
rs1800636:	CC	59 (42)	51	8	44	1.0		0.3183	1.0		0.2561
	CT	57 (41)	46	11	42	1.52 (0.56-4.12)	0.406		0.95 (0.42-2.19)	0.913	
	TT	23 (17)	22	1	13	0.29 (0.03-2.46)	0.256		0.44 (0.16-1.22)	0.115	
rs1800637:	TT	58 (42)	50	8	44	1.0		0.5385	1.0		0.2362
	TC	56 (40)	46	10	41	1.36 (0.49-3.74)	0.553		0.87 (0.37-2.02)	0.746	
	CC	25 (18)	23	2	14	0.54 (0.11-2.76)	0.462		0.40 (0.15-1.09)	0.074	
rs1800639:	GG	59 (42)	51	8	44	1.0		0.3183	1.0		0.2561
	GT	57 (41)	46	11	42	1.52 (0.56-4.12)	0.406		0.95 (0.42-2.19)	0.913	
	TT	23 (17)	22	1	13	0.29 (0.03-2.46)	0.256		0.44 (0.16-1.22)	0.115	
	TT	57 (41)	49	8	44	1.0		0.3183	1.0		0.2561
	TC	57 (41)	46	11	42	1.52 (0.56-1.12)	0.406		0.95 (0.42-2.19)	0.913	
	CC	23 (17)	22	1	13	0.29 (0.03-2.46)	0.256		0.44 (0.16-1.22)	0.115	
	Failed genotyping	2 (1)									
rs2298787:	TT	57 (41)	50	7	42	1.0		0.3208	1.0		0.4143
	TC	59 (42)	47	12	44	1.82 (0.66-5.03)	0.245		1.05 (0.46-2.41)	0.913	
	CC	23 (17)	22	1	13	0.32 (0.04-2.80)	0.306		0.46 (0.17-1.28)	0.138	
rs2578187:	GG	114 (82)	99	15	78	1.0		0.9719	1.0		0.5744
	GA	24 (17)	19	5	20	1.74 (0.56-5.35)	0.336		2.31 (0.74-7.24)	0.152	
	AA	1 (1)	1	0	1	-	-		-	-	
rs2578189:	CC	89 (64)	78	11	67	1.0		0.0014	1.0		0.3898
	CT	45 (32)	38	7	28	1.31 (0.47-3.64)	0.609		0.54 (0.25-1.17)	0.118	
	TT	5 (4)	3	2	4	4.73 (0.71-31.52)	0.109		1.31 (0.14-12.38)	0.812	
rs7244921:	TT	59 (42)	51	8	44	1.0		0.3183	1.0		0.2561
	TC	57 (41)	46	11	42	1.52 (0.56-4.12)	0.406		0.95 (0.42-2.19)	0.913	
	CC	23 (17)	22	1	13	0.29 (0.03-2.46)	0.256		0.44 (0.16-1.22)	0.115	
rs8088881:	TT	58 (42)	50	8	44	1.0		0.3179	1.0		0.2493
	TC	57 (41)	46	11	42	1.49 (0.55-4.04)	0.429		0.89 (0.38-2.07)	0.788	
	CC	23 (17)	22	1	13	0.28 (0.03-2.41)	0.249		0.41 (0.15-1.15)	0.090	
	Failed genotyping	1 (1)									
rs8089150:	AA	57 (41)	50	7	42	1.0		0.3208	1.0		0.4143
	AG	59 (42)	47	12	44	0.82 (0.66-5.03)	0.245		1.05 (0.46-2.41)	0.913	
	GG	23 (17)	22	1	13	0.32 (0.04-2.80)	0.306		0.46 (0.17-1.28)	0.138	
rs8092674:	CC	59 (42)	51	8	44	1.0		0.3183	1.0		0.2561
	CT	57 (41)	46	11	42	1.52 (0.56-4.12)	0.406		0.95 (0.42-2.19)	0.913	
	TT	23 (17)	22	1	13	0.29 (0.03-2.46)	0.256		0.44 (0.16-1.22)	0.115	
rs8093880:	CC	57 (41)	49	8	43	1.0		0.3322	1.0		0.3575
	CT	55 (40)	45	10	42	1.36 (0.49-3.75)	0.551		1.05 (0.44-2.50)	0.909	
	TT	23 (17)	22	1	13	0.28 (0.03-2.36)	0.241		0.42 (0.15-1.18)	0.099	
	Failed genotyping	4 (3)									
rs8094902:	TT	59 (42)	51	8	44	1.0		0.3183	1.0		0.2561
	TC	57 (41)	46	11	42	1.52 (0.56-4.12)	0.406		0.95 (0.42-2.19)	0.913	
	CC	23 (17)	22	1	13	0.29 (0.03-2.46)	0.256		0.44 (0.16-1.22)	0.115	
rs8095031:	TT	59 (42)	51	8	44	1.0		0.3234	1.0		0.2221
	TC	57 (41)	46	11	42	1.52 (0.56-4.12)	0.406		0.95 (0.42-2.19)	0.913	
	CC	22 (16)	22	1	12	0.30 (0.04-2.58)	0.275		0.41 (0.15-1.14)	0.087	
	Failed genotyping	1 (1)									
rs8095608:	AA	58 (42)	51	7	43	1.0		0.3183	1.0		0.3738
	AG	58 (42)	46	12	43	1.90 (0.68-5.24)	0.214		1.0 (0.44-2.30)	1.000	
	GG	22 (16)	21	1	12	0.35 (0.04-2.99)	0.336		0.42 (0.15-1.17)	0.096	
	Failed genotyping	1 (1)									

*Log-Rank p-value.

Demographics of the genotype frequencies in Australian FAP patients with CRC, patients with adenomas and cancer/adenoma free individuals. Logistic regression model was used to test risk of CRC associated with each SNP, while Kaplan-Meier estimator analysis was used to test association between age of diagnosis of CRC/adenomas and genotype. Two different analysis was performed; one using CRC as endpoint of analysis and one using adenomas as endpoint of analysis.

### Validation cohort

To confirm the significant association observed in the Australian FAP cohort for SNP rs2578189 we recruited an independent Dutch FAP cohort from Leiden University Medical Centre. Genotyping of rs2578189 was outsourced to KBioscience (England, UK) who genotyped 427 FAP patients with a germline *APC* mutation from 182 Dutch families with a KASP assay. Due to extensive follow-up and surveillance of this FAP cohort, we were able to divide the patients into two groups, those that developed cancer and those who developed adenomas. Furthermore, we defined AFAP as <100 adenomas and Classic FAP as according to the presence of >100 adenomas.

## Results and discussion

As cancer development in general is known to be influenced by both genetic and environmental factors, it is important to explore the possibility of different genes being involved in modifying the expression of disease in FAP patients - as patients harbouring identical mutations in *APC* can have very different disease profiles that cannot be explained by environmental factors alone. Identification of FAP patients with a modifying genotype is important for the implementation of screening strategies at an early age to remove premalignant adenomas that are likely to be associated with disease development. As it has been suggested that raised levels of *ATP5A1* expression have been linked to certain SNPs and high expression levels may facilitate CRC development [28], we aimed to study several SNPs in this gene to determine whether they influence the age of diagnosis or risk of developing CRC.

### Association testing between disease severity and *ATP5A1*

The genotype frequency distribution for the different SNPs in this study was determined to establish a correlation with disease development in FAP patients. All sixteen SNPs were in Hardy-Weinberg equilibrium (HWE). Genotype frequency of the sixteen SNPs, results of simple logistic regression and Kaplan-Meier analysis can be seen in Table 1. In summary, an association between age of diagnosis of CRC and genotypes can be seen for SNP rs2578189 (Log-rank *p* = 0.0014, Wilcoxon *p* = 0.0004 and Tarone-Ware *p* = 0.0009) with individuals harbouring the variant genotype (TT) developing CRC 29 years earlier than patients harbouring the wildtype genotype (CC), median age of diagnosis of CRC is 74 years for genotype CC, 55 years for genotype CT and 45 years for genotype TT. Even though logistic regression did not show a significant influence of this SNP on risk of developing CRC (see Table 1), a multiple logistic regression was performed to adjust for gender and mutation group; a trend towards the variant genotype increasing your risk of CRC was observed (HR = 7.85, 95% CI = 0.86-71.89, *p* = 0.068), which seems to be influenced by mutation group (*p* = 0.028) and not gender (*p* = 0.296).

Cox proportional hazard regression (adjusted for gender and mutation group) further demonstrates that individuals harbouring the variant genotype of SNP rs2578189 are at higher instantaneous risk (increased hazard) compared to individuals harbouring the wildtype genotype (hazard ratio (HR) = 13.79, 95% CI = 2.36-80.64, *p* = 0.004). Our results indicate that the variant genotype of SNP rs2578189 in the *ATP5A1* gene is a possible modifier of cancer development in Australian FAP patients. We therefore recruited an independent Dutch FAP cohort for validation analysis.

### Validation studies of the observed association

The results from the independent Dutch FAP cohort for SNP rs2578189 can be seen in Table 2. The association between age of diagnosis of CRC and SNP rs2578189 genotypes seen in the Australian FAP cohort was not observed in the Dutch FAP cohort. Neither is the increased risk of CRC observed with Cox proportional hazard regression when the variant genotype of SNP rs2578189 is compared to the wildtype genotype: CRC as endpoint of analysis (HR = 0.91, 95% CI = 0.15-5.65, *p* = 0.915) or adenomas as endpoint of analysis (HR = 0.98, 95% CI = 0.42-2.32, *p* = 0.973).

**Table 2:** Logistic regression and Kaplan-Meier analysis

**Logistic regression model (n = 423)**						**Analysis endpoint - CRC**				**Analysis endpoint - Adenomas**			
***APT5A1 *** **SNP: Genotype**		**Total n (%)**	**Cancer free (n)**	**CRC (n)**	**Adenomas (n)**	**OR (95% CI)**	**p-value**	Kaplan-Meier analysis p-value*	**OR (95% CI)**	**p-value**	Kaplan-Meier analysis p-value*		
rs2578189:	CC	325 (77)	287	38	313	1.0		0.8739	1.0		0.5916
	CT	89 (21)	81	8	87	0.75 (0.33-1.66)	0.473		1.53 (0.33-70.3)	0.585	
	TT	9 (2)	7	2	8	2.16 (0.43-10.77)	0.348		0.28 (0.03-2.45)	0.250	

*Log-Rank p-value.

Demographics of the genotype frequencies in Dutch FAP patients with CRC, patients with adenomas, and cancer/adenoma free individuals. Logistic regression model was used to test risk of CRC associated with each SNP, while Kaplan-Meier estimator analysis was used to test association between age of diagnosis of CRC/adenomas and genotype. Two different analyses were performed; one using CRC as endpoint of analysis and one using adenomas as endpoint of analysis.

The biggest difference between the Australian and Dutch FAP cohort is the extensive follow-up data from the Netherlands about adenoma counts and surgical intervention (284/427 Dutch patients have had surgery). As surgery does not fully protect against CRC and adenoma-category and mutation group are known to influence disease development, we added these confounding factors as co-variats (plus gender as this was done for the Australian cohort) in the Cox proportional hazard regression model for SNP rs2578189 but this did not change the outcome of the analysis (CRC as endpoint of analysis; HR = 0.65, 95% CI = 0.11-3.98, *p* = 0.639 or adenomas as endpoint of analysis; HR = 0.96, 95% CI = 0.41-2.28, *p* = 0.929).

### Validation cohort – disease expression

**Figure 1:**
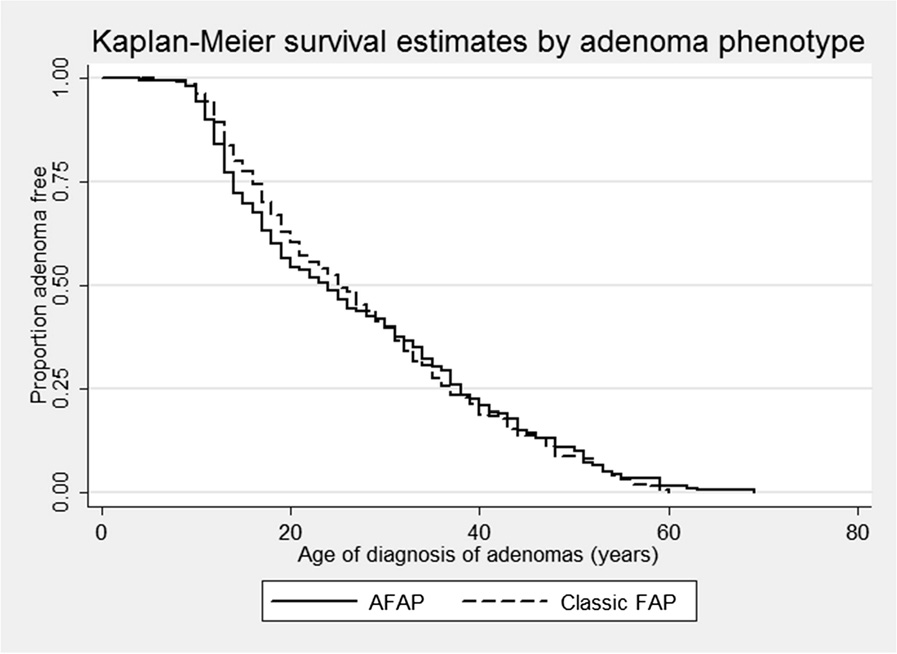
**Kaplan-Meier estimated by adenoma phenotype using adenomas as end-point of analysis.** The graph shows the effect the adenoma phenotype has on age of diagnosis of adenomas in Dutch FAP patients. The adenoma-categories are: AFAP = <100 adenomas and Classic FAP = 100 or more adenomas. No significant difference can be seen between adenoma phenotype AFAP (24 years) and Classic FAP (25 years); Log-rank *p* = 0.8923.

**Figure 2:**
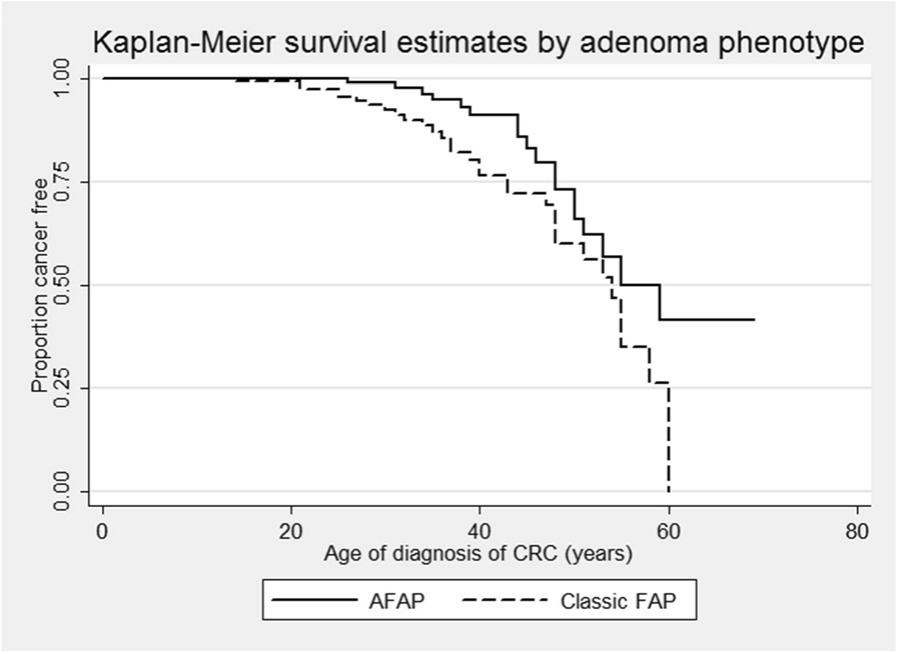
**Kaplan-Meier estimated by adenoma phenotype using CRC as end-point of analysis.** The graph shows the effect the adenoma phenotype has on age of diagnosis of CRC in Dutch FAP patients. The adenoma-categories are: AFAP = <100 adenomas and Classic FAP = 100 or more adenomas. A significant difference is observed between adenoma phenotype and age of diagnosis of CRC (Log-rank *p* = 0.0256), even though there is no difference between ages of diagnosis of CRC and adenoma phenotypes can be seen when 50% of the population is cancer free (AFAP = 55 years and Classic FAP = 54 years). An increased risk of CRC for patients in the Classic FAP compare to AFAP phenotype is observed: OR = 2.12, 95% CI = 1.14-3.92 and *p* = 0.017).

As shown before [7-9,11], phenotypic variability exists within families with FAP suggesting that beside the *APC* genotype other factors also play a role in determining the severity of polyposis and risk of CRC for FAP patients. Even though no significant difference can be seen between age of diagnosis of adenomas and adenoma phenotype (AFAP vs. Classic FAP; log-rank *p* = 0.892, see Figure 1), the risk of CRC could still be influenced by the number of adenomas. We observed a significant difference between age of diagnosis of CRC and adenoma phenotype, see Figure 2 (Log-rank *p* = 0.0256, Wilcoxon *p* = 0.0083 and Tarone-Ware *p* = 0.0124), with individuals developing more adenomas (Classic FAP, 100 or more adenomas) developing CRC earlier than AFAP (<100 adenomas). Logistic regression shows that individuals with Classic FAP phenotype are at increased risk of CRC compared to AFAP phenotype; OR = 2.12, 95% CI = 1.14-3.92, *p* = 0.017. This is in accordance with previously reported results where an increased risk of CRC and earlier ages of diagnosis is associated with greater number of adenomas [9,11,22]. We also adjusted this analysis according to gender, surgery and mutation group of the patients, as this are all confounding factors that can possibly influence an individual’s risk of developing CRC. Multiple logistic regression model did not display an increased risk of CRC (OR = 0.90, 95% CI = 0.46-1.77, *p* = 0.764), but shows that patients who have had interventional surgery are at increased risk of CRC (OR = 14.83, 95% CI = 3.34-65.91 and *p* ≤ 0.001) indicating that one adenoma phenotype has had more surgery than the other group, which is correct as 94% of Classic FAP patients have had surgery vs. 42% of AFAP patients. Simple (HR = 1.78, 95% CI = 0.88-3.62, *p* = 0.107) and multiple (HR = 1.14, 95% CI = 0.56-2.35, *p* = 0.706) Cox proportional hazard regression similarly display no significant increased risk of CRC according to adenoma phenotype, but again demonstrates that surgical intervention (*p* = 0.015) are a marker for the more severe polyposis phenotype (Classic FAP). This is also the case for mutation group; APC MCR (*p* ≤ 0.001).

**Figure 3:**
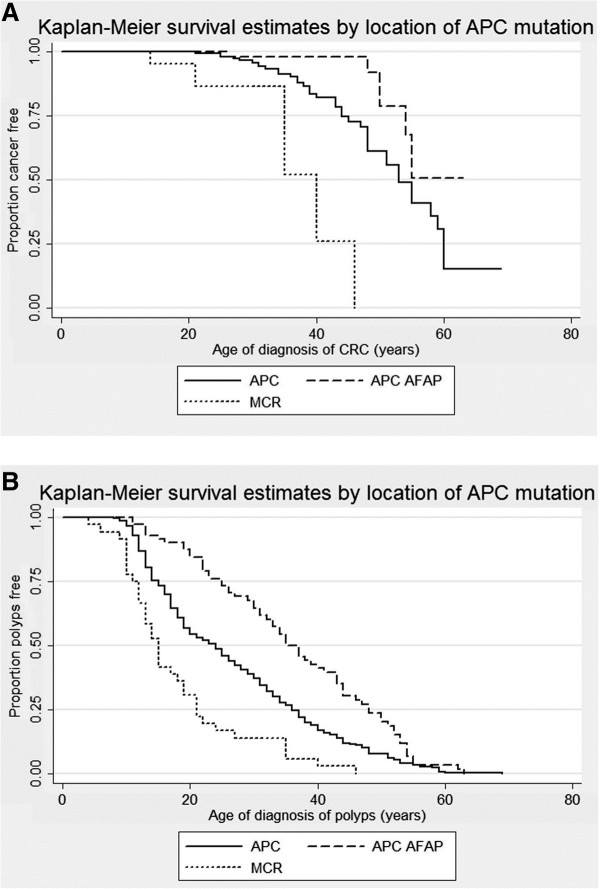
**Kaplan-Meier estimated by location of APC mutation. A)** Using CRC as end-point of analysis: The graph shows the effect the location of the *APC* mutation has on age of diagnosis of CRC in Dutch FAP patients. A significant difference is observed between the attenuated (APC AFAP), intermediate (APC, 53 years) and severe (MCR, 40 years) polyposis phenotype; Log-rank *p* ≤ 0.0001. **B)** Using adenomas as end-point of analysis: The graph shows the effect the location of the *APC* mutation has on age of diagnosis of adenomas in Dutch FAP patients. A significant difference is observed between the attenuated (APC AFAP, 35 years), intermediate (APC, 24 years) and severe (MCR, 15 years) polyposis phenotype; Log-rank *p* ≤ 0.0001.

The severity of polyposis and early onset CRC is dependent on the location of *APC* mutations [7,13]. The Dutch FAP patients with mutations in the APC MCR develops CRC 13 years earlier than patients with mutations in the rest of the *APC* gene, while patients with mutations in APC AFAP regions develops CRC later compared to individuals with mutations in the rest of the *APC* gene (log-rank *p* ≤ 0.0001, see Figure 3A). The same is observed when adenomas is used as endpoint of analysis with patients developing adenomas at ages; APC AFAP = 35 years, APC = 24 years and MCR = 15 years (log-rank *p* ≤ 0.0001, see Figure 3B). Cox proportional hazard regression also shows an increased risk of CRC and adenomas for patients with mutations in APC MCR compared to patients with mutations in the rest of the *APC* gene (APC mutation category): CRC (HR = 7.35, 95% CI = 2.44-22.14 and *p* ≤ 0.001) and adenomas (HR = 2.36, 95% CI = 1.54-3.61 and *p* ≤ 0.001). We also adjusted this analysis for gender, surgery and adenoma phenotype – the results are still significant; CRC (HR = 7.71, 95% CI = 2.53-23.49 and *p* ≤ 0.001) and adenomas (HR = 2.71, 95% CI = 1.72-4.29 and *p* ≤ 0.001) and are influenced by whether the patients have had surgery (CRC *p* = 0.015 and adenomas *p* ≤ 0.001), not gender (CRC *p* = 0.869 and adenomas *p* = 0.923) or adenoma phenotype (CRC *p* = 0.706 and adenomas *p* = 0.055). Not surprisingly, this is showing us that interventional surgery is the biggest marker for patients with mutations in APC MCR and are therefore at higher risk of CRC. As expected, a decreased risk of CRC and adenomas is observed for patients with mutations in APC AFAP regions compared to patients with mutations in the rest of the *APC* gene: CRC (HR = 0.28, 95% CI = 0.10-0.78 and *p* = 0.015) and adenomas (HR = 0.49, 95% CI = 0.33-0.73 and *p* ≤ 0.001).

The risk of developing CRC in FAP patients is considerably different from the most common genetic predisposition to CRC, Lynch syndrome, with earlier ages of disease onset and close to 100% penetrance if left untreated. Lynch syndrome cases that are not under surveillance are usually identified when they present with CRC whereas FAP patients initially present with adenomas that are removed before the development of CRC and often undergo total colectomy to reduce the risk of cancer development. The timing of surgery may have influenced adenoma counts such that some patients may have, if left untreated, developed many more adenomas than that identified at colectomy. For this reason, it is much more difficult to study CRC risk and differences in the phenotypic expression in FAP patients as many of them will have interventional surgery to reduce their risk of developing CRC. This could possibly explain the differences observe between the Dutch and Australian patient data, as we have access to extensive follow-up and surveillance data from the Dutch FAP patients that are not available for the Australian patients.

*Atp5a1* in mice is located on the same chromosome as *Apc*, linked in the *cis* configuration on chromosome 18. The modifier locus acts in a dominant fashion to markedly reduce intestinal polyp multiplicity in mice with *Apc* mutations [26]. One of the reasons why we cannot conclusively associate *ATP5A1* in FAP patients with adenoma counts or disease risk could be that in humans *APC* is located on chromosome 5, while *ATP5A1* is located on chromosome 18.

## Conclusion

These results highlight the difficulties in studying a disease that has a high degree of intervention and also emphasize the importance of large sample sizes when searching for modifier genes in patients with an inherited predisposition to disease. To fully determine if there are genetic modifiers of disease in FAP we would encourage people that are interested in collaborating in future studies into the role of modifier genes in disease expression in FAP to join forces. Even though we were not able to associate SNPs of *ATP5A1* with FAP, there remains the possibility that there are genetic modifiers that influence disease severity in FAP due to the differences observed in disease expression.

## Competing interests

The authors declare that they have no competing interests.

## Authors’ contributions

BTP: Study design; acquisition of data; analysis and interpretation of data; drafting of the manuscript; statistical analysis. JTW: Acquisition of data; analysis and interpretation of data; critical revision of the manuscript for important intellectual content; technical support; obtained funding; study supervision. EKA: Acquisition of data; analysis and interpretation of data. DB: Statistical advice; analysis and interpretation of data. SJC: Acquisition of data. CMT: Acquisition of data; material support. CM: Acquisition of data; material support. The Dutch Cancer Genetics Group: Patient collection. AS: Patient collection; material support. FJH: Acquisition of data; critical revision of the manuscript for important intellectual content. TVW: Acquisition of data; critical revision of the manuscript for important intellectual content. HFAV: Study concept and design; critical revision of the manuscript for important intellectual content. RJS: Study concept and design; critical revision of the manuscript for important intellectual content; obtained funding; study supervision. All authors read and approved the final manuscript.
